# Adapting to the health impacts of climate change in a sustainable manner

**DOI:** 10.1186/s12992-014-0082-8

**Published:** 2014-12-11

**Authors:** Damian Hoy, Adam Roth, Christelle Lepers, Jo Durham, Johann Bell, Alexis Durand, Padma Narsey Lal, Yvan Souares

**Affiliations:** Public Health Division, Secretariat of the Pacific Community, BP D5 - 98848 Noumea, New Caledonia; The University of Queensland, Faculty of Medicine & Biomedical Sciences, School of Population Health, Herston Road, 4006 Herston, Qld Australia; Visiting Professorial Fellow, Australian National Centre for Ocean Resources and Security, University of Wollongong, 2252 Wollongong, NSW Australia; Brown University, Brown Street, Box 7178 69, 02912 Providence, RI USA; CSIRO (Land and Water Division, Black Mountain), Clunes Ross Street, Action, ACT 0200 Canberra City, Australia

**Keywords:** Climate, Change, Adaptation, Health, Resilience, Development effectiveness, Harmonisation, Alignment

## Abstract

The climate is changing and this poses significant threats to human health. Climate change is one of the greatest challenges facing Pacific Island countries and territories due to their unique geophysical features, and their social, economic and cultural characteristics. The Pacific region also faces challenges with widely dispersed populations, limited resources and fragmented health systems. Over the past few years, there has been a substantial increase in international aid for health activities aimed at adapting to the threats of climate change. This funding needs to be used strategically to ensure an effective approach to reducing the health risk from climate change. Respecting the principles of development effectiveness will result in more effective and sustainable adaptation, in particular, 1) processes should be owned and driven by local communities, 2) investments should be aligned with existing national priorities and policies, and 3) existing systems must not be ignored, but rather expanded upon and reinforced.

## Introduction

The earth’s surface has warmed by 0.6 degrees Celsius over the past 30 years, and this is estimated to increase further (between 1.1 and 6.4 degrees Celsius) this century [1]. Climate change poses significant threats to human health [2]. Anticipated impacts include 1) food shortages as a result of lower yields of crops and fisheries in some locations, 2) reduced access to clean water due to changing rainfall patterns, 3) increased incidence of malaria, dengue, influenza, heatstroke and other climate-sensitive diseases due to rising temperatures, and 4) injury, illness and death from weather-related disasters [1,3-7].

Climate change is one of the greatest challenges facing Pacific Island countries and territories (PICTs) due to their unique geophysical features and their social, economic and cultural characteristics [8,9]. Life expectancies in the Pacific are generally low [10] and, in many countries, have not improved significantly over the last 20 years [11]. Most Pacific communities have a high prevalence of non-communicable diseases, and some also have a high burden from communicable diseases such as tuberculosis and dengue fever [11,12]. These challenges are compounded by widely dispersed populations, limited resources and fragmented health systems, which are highly reliant on development funding.

Adaptation activities involve putting in place measures that reduce the vulnerability of human or natural systems to climate change impacts [1,13]. To provide an example, the changing climate may result in an increase in the incidence of dengue [14-16]. Adaptations may include strengthening the resilience and capacity of ministries of health and environment, as well as the general community, in routine vector control to minimise the presence of potential mosquito breeding sites, disease surveillance systems to detect dengue outbreaks, and response mechanisms to help ensure dengue outbreaks are promptly brought under control to minimise morbidity, mortality and health system strain.

There has been a substantial increase over recent years in international development funding for adaptation activities in the health sector [17]. While this is much needed, if it is not used according to principles of development effectiveness, it has the potential to produce a multitude of stand-alone climate change projects and programmes, thereby increasing fragmentation within the health sector and other sectors [18]. Given the potential effects of climate change are multi-sectoral, adaptation to the threats of climate change should be implemented alike. In this paper, we discuss how climate change and health development activities in the Pacific can be delivered in an effective and efficient way to help ensure positive, sustainable outcomes.

## What are the key principles of development effectiveness?

International agreements on development effectiveness aim to improve the quality and effectiveness of development cooperation [18-20]. The Paris Declaration on Aid Effectiveness emphasised the importance of ownership, alignment and harmonisation [20]. Following this, the Accra Agenda for Action stressed the importance of country ownership, reducing fragmentation, and improving coordination through effective and inclusive partnerships [19]. The Busan Partnership for Effective Development Cooperation re-emphasised principles of ownership, inclusive development partnerships, reducing fragmentation and the number of aid mechanisms, and alignment with developing country priorities and policies. These agreements have been adapted to the Pacific through the Pacific aid effectiveness principles (2007) [21], and the Cairns Compact on Improving Development Coordination [22]. Key principles included are country ownership, accountable and transparent planning and financial systems, alignment with nationally identified priorities, coordination and harmonisation of development assistance, and increased use of local systems by development partners.

## How can the principles of development effectiveness be utilised in climate change and health adaptation?

### Community ownership and inclusive development partnerships

Inclusion and empowerment of local communities is vital at every stage of a development initiative. Planning and decision-making processes should be locally-owned, and extensive community consultation should take place to facilitate this ownership. Community consultation also invariably demonstrates that much of the expertise required for adaptation is available at the local level. While expertise from regional or international organisations may be beneficial, and useful in sharing lessons from other countries, any external support should always aim to build local adaptive capacity [23].

The Pacific Public Health Surveillance Network (PPHSN) is one such body that is designed for the purpose of improving local ownership as well as more inclusive development partnerships [24]. It was established in 1996 and is a voluntary network of countries, areas and organizations, dedicated to the promotion of public health surveillance and response in the Pacific. The core members are the 22 Ministries and Departments of Health in the Pacific Island countries and territories, and whose authority or consensus guides its work [25]. The PPHSN is further supported by allied bodies — regional and international agencies, training institutions, laboratories, and other partners who can supplement the network with technical expertise, and in-kind and financial support. A Coordinating Body (CB) is drawn from each of these two groups, and consists of seven core and five allied body members. The CB membership is rotated in a staggered manner, with membership voted or endorsed by network and CB members. Three members are permanent (SPC, WHO and the Fiji National University), and one (SPC) has been designated Focal Point. The additional two allied members of the current CB are PIHOA and CDC.

The Pacific Data for Decision Making (DDM) Program, which is an accredited training program through Fiji National University, is implemented at the national level throughout the Pacific by PPHSN partners. The aim of the Program is to strengthen the capacity of PICTs to be able to utilize disease and syndromic data, including from climate-sensitive diseases and syndromes, for responding to outbreaks and broader decision-making. The Program is in response to the Pacific Health Ministers’ recommendation *“to address the lack of trained and experienced epidemiologists in the region...... development of comprehensive training programmes to develop core competencies in “data techs”, “epi techs” and epidemiologists”* [26]. Consequently, there is a heightened sense of local ownership over this Program, which is also reinforced by having local facilitators, and by participants bringing their own real data to the trainings.

Further consultation has been undertaken for the DDM Program through a number of additional initiatives, including national climate change and health vulnerability assessments, and the 2012 Pacific Regional Climate Change and Health Symposium (PRCCHS). The symposium was hosted by the University of Fiji and supported by the Secretariat of the Pacific Community (SPC), the World Health Organisation (WHO) and the Fiji Ministry of Health. The event brought together over 130 key stakeholders from a number of PICTs, as well as Australia, New Zealand, Japan and Korea. They included researchers, academics, policy-makers, representatives from government and non-government sectors, and students. The PRCCHS Outcome Statement highlighted *“the need to strengthen health information and surveillance systems for better decision-making, including for health adaptation. A key focus is the need to build local capacity in the analysis and use of existing information*” [5]. This further reinforced the sense of Pacific ownership over the Pacific Data for Decision Making Program. Broad consultation continues to be undertaken to ensure a constant and appropriate improvement cycle.

### Alignment

Adaptation initiatives need to be integrated with existing national priorities and policies [18]. This includes indicators for monitoring and evaluating the performance of adaptation initiatives [18]. Consulting and collaborating broadly with government and non-government bodies across a variety of sectors, such as environment, education, employment, transport, infrastructure, agriculture, fisheries and housing, will help facilitate the alignment of new endeavours to existing initiatives. This will also promote integration; help avoid duplication; improve ownership, participation and commitment; and provide opportunities to share resources [20,27,28]. Vanuatu, for example, is aligning and integrating their climate change agenda and their water, sanitation, and hygiene strategies into programmatic activities across departments, such as communications and education, and the National Health Disaster Plan [29]. This is likely to result in broad benefits to all of these sectors.

### Harmonisation, coordination and use of local systems

Climate change influences and is influenced by every sector in society, so collaboration between different government sectors, institutions and communities is required for effective adaptation [3]. The Outcome Statement from the 2012 PRCCHS recognised *“that ‘climate change and health’ is a domain that is multi-sectoral and this consequently needs a broad range of people involved with skills, experience and expertise from all sectors and from grassroots through to national, regional and global levels”* [5]. The Statement also encourages *“mainstreaming climate change and health issues into formal curricula at the primary, secondary and tertiary levels in health and other training institutions”* [5].

In the Pacific, the national vulnerability assessments on the health impacts of climate change informed the development of National Climate Change and Health Action Plans (NCCHAPs). These NCCHAPs are highly dependent on donor funding if they are to be implemented; however, if they are implemented according to principles of development effectiveness, they have great potential to promote more harmonised national and regional health systems. It is critical that all agencies involved in climate change and health adaptation initiatives collaborate closely. Having both internal and external partners assessed for their collaborative capacity, including their ability and willingness to collaborate with other players, has been discussed recently by Lai, and is worthy of serious consideration for the Pacific [30].

Climate change adaptation initiatives, wherever possible, should be conducted through existing national and regional systems and processes [31]. It is important to ensure that these existing systems are built upon and strengthened rather than ignored. All stakeholders have a responsibility to conduct thorough consultations before embarking on any development activity, including those relating to climate change and health. Part of this consultation should assess what systems are in existence. Regionally, a number of systems and networks are currently in existence, including the PPHSN, and the Pacific Health Information Network (PHIN). Table 1 gives an example of some needs identified in NCCHAPs and demonstrates what existing regional initiatives are in place to help support these activities.

**Table 1:** **Examples of needs identified in National Climate Change and Health Action Plans and what existing regional initiatives are in place to help support these activities**

**PICT**	**Example of key NCCHAP recommendations**	**Examples of key existing regional initiatives**
Niue	Improve HIS capacity, including data collection, coding, storage and analysis	● Pacific data for decision-making program
	Improve communication and collaboration between agencies to streamline activities, share information and resources and avoid duplication	● Pacific Public Health Surveillance Network
		● Pacific Health Information Network
Palau	Build capacity in the following areas: reporting, coding, storage, analysis and dissemination of health data; recruitment and training of health personnel in biostatistics and epidemiology; public health, primary health care, environmental health and laboratory facilities; health research, including public and environmental health.	● Pacific data for decision-making program
		● Pacific Public Health Surveillance Network – EpiNet, LabNet
		● Pacific Operational Research Course
Republic of the Marshall Islands	Improve health information/data collection system, including: improve current biostatistics reporting system; assure ongoing quality control; recruit and/or train extra personnel in environmental health (including vector-borne disease control), biostatistics and epidemiological analysis	● Pacific data for decision-making program
		● Pacific Public Health Surveillance Network – EpiNet, LabNet

Climate change and health should not be viewed as a vertical programme, but rather one that expands across the entire health system and beyond. The real and potential negative impacts of taking a vertical approach to health system strengthening in low- and middle-income countries are well established. These include difficulties coordinating resources and activities, duplication of activities, loss of staff to vertical programs due to better pay, and inequalities created by focusing on vertical programs at the detriment of other important services [32-35]. More than anywhere in the world, Pacific health systems cannot afford the luxury of vertical programs; the structurally limited human resources, largely resulting from tiny populations, within these systems are simply unable to meet the demand of such approaches. Integration of climate change and health across the health system is an issue that requires careful thought and extensive consultation.

Therefore, efforts to strengthen capacity and systems for climate-sensitive disease surveillance and response should take a system-wide approach. This will have several benefits, including: 1) ensuring that climate change initiatives are well-integrated and embedded within existing systems and thus more likely to be sustained; 2) ensuring that existing systems are built upon rather than duplicated resulting in improved ownership and more effective investment; 3) helping to address the high levels of staff turnover in the Pacific by developing a cadre of health surveillance and response generalists who are equipped to respond to changing environments; and 4) ensuring that other aspects of the health system are not neglected.

Information gathering, analysis and dissemination are priorities for the public health community in PICTs, especially in light of increasing risks posed by long-term global changes. Flexible and robust information systems can inform priority setting, the design of evidence-based interventions, the evaluation of outbreak response, and the creation of early warning systems. To date, however, health information systems in many PICTs have been limited and fragmented, and variable data content and quality have made it difficult to capture long-term trends in the region.

All PICTs conduct public health surveillance activities and submit data to regional and international agencies. Each country or territory has its own system of data collection, processing, and reporting. Key challenges are that there is heavy demand on PICTs to provide information on a large number of indicators, which are largely determined by external agencies, and these indicators are usually requested in formats prescribed differently by each of the agencies [24].

Further, training programs for data collection, analysis and interpretation at the national level have traditionally not always been coordinated between regional and global partners. The countries and territories concede that it is difficult to provide quality data with the high level of demands upon them, and that the data are therefore of limited usefulness in local and regional decision-making and action. One of the key functions of the PPHSN is to address these challenges. One of the mechanisms for this is through strengthening national syndromic surveillance systems in the Pacific. The WHO, SPC and other PPHSN partners have been working over a number of years to achieve this [25].

To complement this work, the Pacific Data for Decision-Making program assesses and develops harmonised plans to improve national syndromic surveillance systems. This work is undertaken at DDM courses with regional PPHSN partners and PICT Ministries and Departments of Health. Further work is needed to improve harmonisation of health and development activities in the Pacific; the PPHSN provides an important avenue for this to happen. The final point of the 2012 PRCCHS Outcome Statement highlights this need: *“We recognise the important need for coordination of climate change and health research and activities across the Pacific to avoid duplication and to minimise the burden placed on local communities”* [5]*.*

## Conclusion

The climate is changing and this will have potentially significant health effects. Over the past few years, there has been a substantial increase in international aid funding for health activities aimed at adapting to the threats of climate change. This funding needs to be used strategically to ensure an effective and efficient approach to reducing the health risks from climate change. Adaptation is likely to be best achieved by adhering to the principles of development effectiveness, including ensuring that processes that are owned and driven by local communities, mainstreamed across the health sector and other sectors, and well aligned to existing programmes. In the Pacific more than anywhere, it is critical than these principles are adopted.
